# Identification of an antibody fragment specific for androgen-dependent prostate cancer cells

**DOI:** 10.1186/1472-6750-14-81

**Published:** 2014-09-03

**Authors:** Ryan M Williams, Cyrus J Hajiran, Sara Nayeem, Letha J Sooter

**Affiliations:** 1 Department of Basic Pharmaceutical Sciences, West Virginia University, 1 Medical Center Drive, PO Box 9530, Morgantown, WV 26506, USA; 2 Current address: Memorial Sloan Kettering Cancer Center, Molecular Pharmacology & Chemistry Program, 1275 York Ave., New York, NY 10065, USA; 3 Department of Biology, West Virginia University, 53 Campus Drive, PO Box 6057, Morgantown, WV 26506, USA

**Keywords:** Prostate cancer, Antibody fragment, scFv, Library screening, Yeast

## Abstract

**Background:**

Prostate cancer is the most-diagnosed non-skin cancer among males in the US, and the second leading cause of cancer-related death. Current methods of treatment and diagnosis are not specific for the disease. This work identified an antibody fragment that binds selectively to a molecule on the surface of androgen-dependent prostate cancer cells but not benign prostatic cells.

**Results:**

Antibody fragment identification was achieved using a library screening and enrichment strategy. A library of 10^9^ yeast-displayed human non-immune antibody fragments was enriched for those that bind to androgen-dependent prostate cancer cells, but not to benign prostatic cells or purified prostate-specific membrane antigen (PSMA). Seven rounds of panning and fluorescence-activated cell sorting (FACS) screening yielded one antibody fragment identified from the enriched library. This molecule, termed HiR7.8, has a low-nanomolar equilibrium dissociation constant (K_d_) and high specificity for androgen-dependent prostate cancer cells.

**Conclusions:**

Antibody fragment screening from a yeast-displayed library has yielded one molecule with high affinity and specificity. With further pre-clinical development, it is hoped that the antibody fragment identified using this screening strategy will be useful in the specific detection of prostate cancer and in targeted delivery of therapeutic agents for increased efficacy and reduced side effects.

## Background

The overall breadth of prostate cancer and problems associated with it render it necessary to develop novel therapeutics and diagnostics for the disease. Prostate cancer is the most-diagnosed non-skin cancer in the United States, with an estimated 233,000 new diagnoses in 2014 alone [1]. It is the second leading cause of cancer-related deaths among males in the U.S., with an estimated 29,480 mortalities in 2014 [1]. The scope of this disease portends the necessity in developing improved clinical tools for its treatment.

It is necessary to develop specifically targeted therapeutics and diagnostics to further aid in treatment of prostate cancer. Current practices for prostate cancer are partially effective; however they are not specific for the disease, causing many unwanted side effects and over-diagnoses. Therapeutic side effects can be serious and leave the possibility for recurrence in a more aggressive, androgen-independent form [2-4]. For many non-localized cancers, chemotherapies are used which can be effective, however specific delivery would be more effective and cause fewer side effects [5-8]. In addition to non-specific therapeutics, prostate cancer diagnosis using prostate specific antigen (PSA) blood levels is no longer recommended for use by the United States Preventive Services Task Force (USPSTF) [9-13]. This is because serum PSA levels are raised not only due to cancerous prostates, but due to benign prostatic conditions such as prostatic intraepithelial neoplasia (PIN), benign prostate hyperplasia (BPH), and prostatitis [14]. Studies have shown the specificity of the PSA test in prostate cancer diagnosis to be just 24%, meaning there is a 76% over-diagnosis rate, and that it will prevent just one prostate cancer-related death in greater than 1000 men [11,15]. Many other biomarkers identified that may have some diagnostic potential, however are not specific for prostate cancer [16-18]. Therefore, it is necessary to develop molecular targeting mechanisms for clinical use against prostate cancer.

An increasingly popular method to develop disease-specific targeting molecules is through antibody fragment library screening. Previous library screening methodologies have been adapted for use in identifying molecules that bind to whole-cell targets [19,20]. Additionally, screening methods exist for utilizing antibody fragment libraries displayed on bacteriophage, bacteria, and yeast [21-23]. Antibody fragments that bind to cell surfaces have largely been selected from phage-displayed libraries, with examples including those that bind to ovarian, breast, and hepatocellular carcinoma cells [24-26]. It is therefore possible to identify antibody fragments which bind to the surface of prostate cancer cells.

The work described here enriches an antibody fragment library for molecules which bind to androgen-dependent prostate cancer cells. Additionally, multiple stringent negative enrichments using targets to which the antibody fragment should not bind were selected against. This work has identified an antibody fragment which binds to androgen-dependent prostate cancer cells, and not to various benign prostate cells. It utilized a human non-immune single-chain Fragment variable (scFv) library displayed on the surface of *Sacchoromyces cerevisiae*. It is hoped that the obtained scFv will be useful for both specific treatment and diagnosis of prostate cancer and serves as proof-of-principle for future screening of disease-specific antibody fragments.

## Results

### Antibody fragment screening

**Figure 1:**
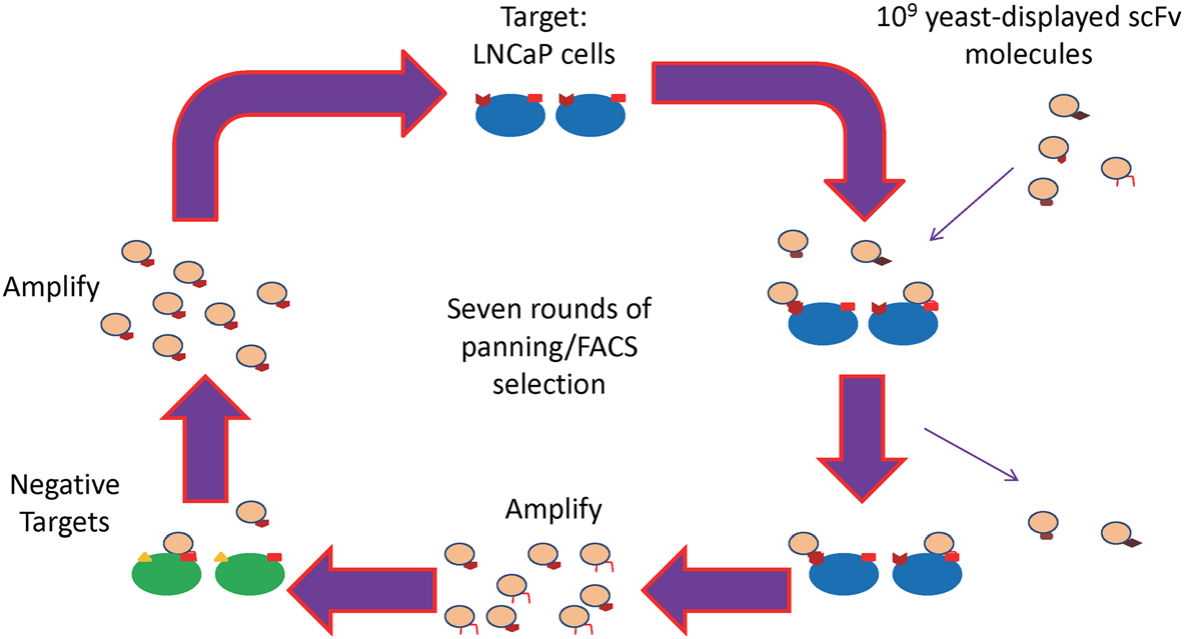
Library screening and enrichment strategy utilized to identify a prostate cancer cell-specific antibody fragment.

In order to obtain androgen-dependent prostate cancer cell-specific antibody fragments, seven rounds of screening and enrichment were completed with a yeast-displayed scFv library [23] (Figure 1, Table 1). Sequencing of a random, representative sample of the scFv-encoding plasmid in the initial naïve library, as well as after Round 2 and Round 3 of panning, was performed. Out of the 30–35 sequences identified, there were no duplicate sequences in any library or between the libraries. This suggests that no particular sequence was selected for in these rounds.

**Table 1:** In vitro selection scheme for selecting an androgen-dependent prostate cancer cell-specific scFv

**Selection round**	**Target**	**Yeast number**	**Human cell number**	**Incubation time (hours)**	**Incubation volume (mL)**	**Separation method**
1 (+)	LNCaP	1 × 10^10^	~8 × 10^6^	3	15	Panning
1 (−)	HGPIN	1 × 10^10^	~7 × 10^6^	0.5	15	Panning
	BPH-1		~9 × 10^6^	0.5		
	BHPrE1		~7.5 × 10^6^	0.5		
2 (+)	LNCaP	1 × 10^10^	~9 × 10^6^	2	15	Panning
2 (−)	HGPIN	1 × 10^10^	~8.5 × 10^6^	1	15	Panning
	BPH-1		~9.5 × 10^6^	1		
	BHPrE1		~8 × 10^6^	1		
3 (+)	LNCaP	1 × 10^10^	~9 × 10^6^	1	15	Panning
3 (−)	HGPIN	1 × 10^10^	~7 × 10^6^	2	15	Panning
	BPH-1		~9 × 10^6^	2		
	BHPrE1		~8 × 10^6^	2		
4 (+)	LNCaP	1 × 10^7^	1 × 10^6^	0.5	2.5	FACS
4 (−) a	HGPIN	1 × 10^7^	1 × 10^6^	0.5	1.5	FACS
4 (−) b	BPH-1	1 × 10^7^	1 × 10^6^	0.5	2	FACS
4 (−) c	BHPrE1	1 × 10^7^	1 × 10^6^	0.5	1.5	FACS
4 (−) d	PSMA	1 × 10^7^	1 nmol protein	0.5	1	FACS
5 (+)	LNCaP	1 × 10^7^	1 × 10^6^	0.5	5	FACS
6 (+)	LNCaP	1 × 10^7^	1 × 10^5^	0.5	2.75	FACS
6 (−) a	HGPIN	1 × 10^7^	1 × 10^6^	0.5	2.5	FACS
6 (−) b	BPH-1	1 × 10^7^	1 × 10^6^	0.5	2.5	FACS
6 (−) c	BHPrE1	1 × 10^7^	1 × 10^6^	0.5	2.5	FACS
6 (−) d	PSMA	1 × 10^7^	1 nmol protein	0.5	2	FACS
7 (+)	LNCaP	1 × 10^7^	1 × 10^3^	0.5	2.5	FACS

**Figure 2:**
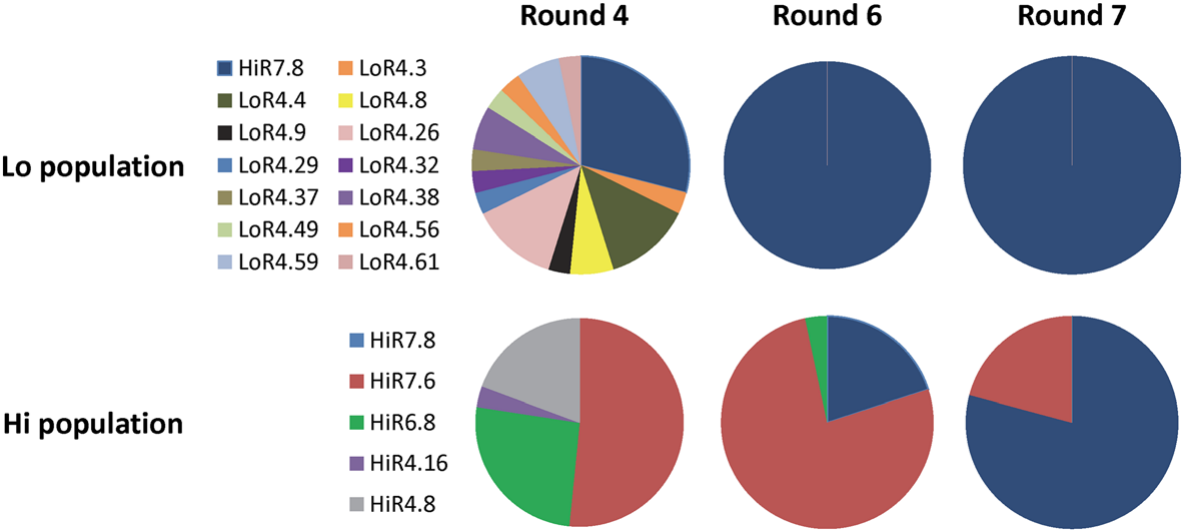
Sequence enrichment in the enriched scFv library in both the Lo and Hi populations beginning in Round 4.

After three rounds, FACS-based screening was performed. In Round 4 (+), there were two separate populations of yeast binding to target cells which were kept separate through the remaining rounds of screening and termed ‘Hi’ and ‘Lo’ (Additional file 1: Figure S1). After Round 4, the Lo population was comprised of 29% HiR7.8 (Figure 2). Interestingly, HiR7.6 and HiR6.8 were not identified in the Lo population. HiR7.8 comprised 100% of the population in Rounds 6 and 7. This suggests that HiR7.8 out-competed all other molecules in the Lo population. It should be noted that HiR7.6 and HiR7.8 are both truncated sequences containing only the heavy chain, while HiR6.8 is a full-length scFv (Table 2).

**Table 2:** Amino acid sequences of HiR6.8, HiR7.6, and HiR7.8

**Sequence name**	**Sequence**	**Predicted molecular weight**
HiR6.8	DVPDYALQASGGGGSGGGGSGGGGS*ASQVQLVQSGAEVKKPGASVKVSCKASGYTFTDYYMHWVRQAPGQGLEWMGWINPNSGGTNYAQKFQGRVTMTRDTSISTAYMELSSLRSDDTAIYYCARDADSGSMSAIYWYFNLWGRGTLVTVSSGILGS*GGGGSGGGGSGGGGS**DIQLTQSPSSLSASVGDRVTITCRASQSISRFLNWYQQKPGKAPKLLIYGGSSLQSGVPSRFSGGGSGTDFTLTISSLQPEDFATYYCQQSYSKFWTFGQGTKVEIKSGIL**EQKLISEEDL	30.6 kDa
HiR7.6	DVPDYALQASGGGGSGGGGSGGGGS*ASQVQLQESGPGLVKPSGTLSLTCAVSGGSISSSNWWSWVRQPPGKELEWIGEIHHSGSTNYNPSLKSRVTISVDKSKNQFSLKMRSVTAADTAVYYCARVEEWPYDALDMWGQGTMVTVSSEF*	15.6 kDa
HiR7.8	DVPDYALQASGGGGSGGGGSGGGGS*ASQVQLQESGPGLVKPSQTLSLTCTVSGDSIYSSGHYWSWVRQHPGKGLEWIGYIYASGRTYYNPSLESRVTMSVDTSKNQSSLKLTSVTAADTAVYYCARDDSRTWYKAFDTWGQGTMVTVSSEF*	16.0 kDa

Italicized amino acids represent the heavy chain. Bold amino acids represent the light chain. HiR7.6 and HiR7.8 do not contain a light chain. Non-italicized or bolded amino acids represent scFv structural elements.

In the Round 4 Hi population, however, HiR7.6 comprised 52% of the population, while HiR6.8 was 26%. Interestingly, HiR7.8 did not appear in the Round 4 Hi population. In the Round 6 population, 77% of the library was HiR7.6 while 3% was HiR6.8. HiR7.8 now comprised 20% of the library. In Round 7, HiR6.8 was no longer detected, while HiR7.6 was now just 16% of the library. HiR7.8 comprised 84% of the library, suggesting it strongly outcompeted HiR7.6. It is also of note that HiR7.6 and HiR7.8 did not appear in any previous library sequencing, however HiR6.8 did appear once in the Round 3 library. This enrichment and convergence upon HiR7.8 validates the sequence enrichment capabilities of this dual-method screening strategy.

### Affinity binding assays of scFvs

**Figure 3:**
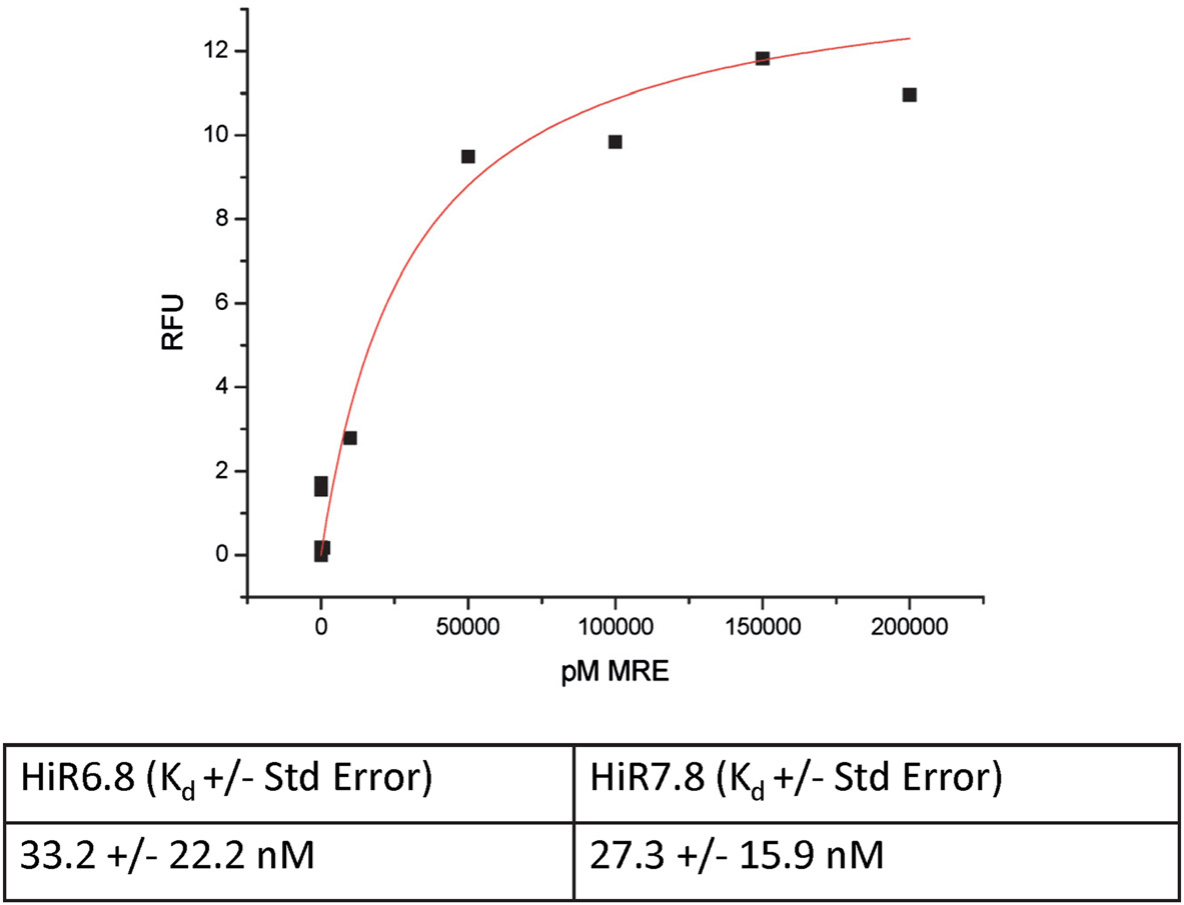
**Equilibrium dissociation constants for HiR7.8 and HiR6.8.** The graph depicts a representative saturation binding curve for HiR7.8 fit with nonlinear regression analysis. The table reports the dissociation constant (K_d_) of both scFvs in nM averaged from three assays with standard errors.

**Figure 4:**
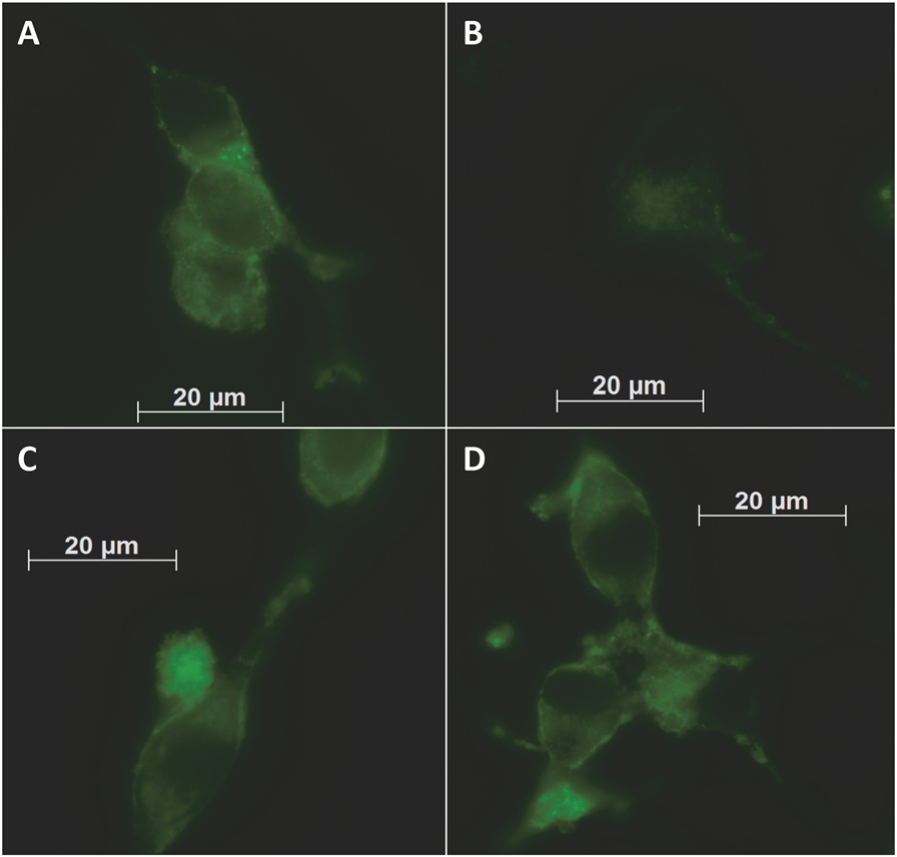
**Representative fluorescent micrographs of LNCaP cells bound by identified scFvs.** Images are at 630X magnification. **(A)** Image of cells tagged with fluorescent anti-PSMA antibody. **(B)** Image of cells incubated with anti-HA secondary fluorescent antibody. **(C)** Image of cells incubated with HiR6.8 and secondary antibody. **(D)** Image of cells incubated with HiR7.8 and secondary antibody.

Once HiR7.8 and HiR6.8 was identified for further characterization, each was cloned into a secretion vector, expressed, purified, and verified by polyacrylamide gel electrophoresis (PAGE). These were used in fluorescent saturation binding assays with LNCaP cells as described in the Methods, plotted, and fit with nonlinear regression analysis (Figure 3). HiR6.8 exhibited an equilibrium dissociation constant (K_d_) of 33.2 +/− 22.2 nM. A K_d_ of 27.3 +/− 15.9 nM was obtained for HiR7.8.Binding of each scFv for androgen-dependent prostate cancer cells is further evidenced by fluorescent micrographic analysis. Binding of each scFv is clear when pre-incubated with a secondary fluorescent antibody and then with target cells and imaged for fluorescent staining (Figure 4C and D). Fluorescence is much brighter than target cells incubated with fluorescent secondary-only control (Figure 4B) and comparable to the positive control (Figure 4A). Interestingly, both antibodies have a horseshoe-like staining pattern for each cell. Therefore, fluorescence may be visualized in the cytosol surrounding the nucleus.

### Specificity binding assays of scFvs

**Figure 5:**
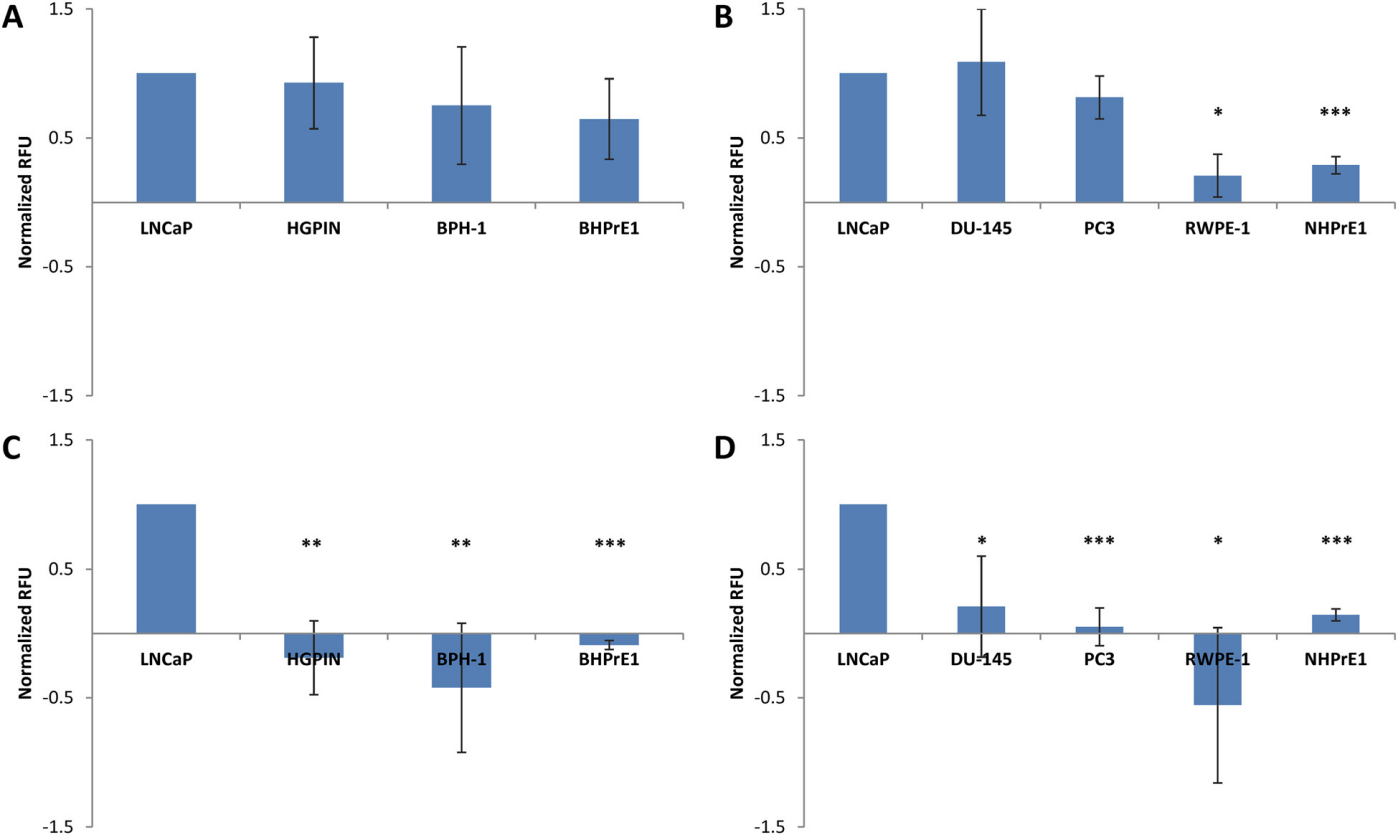
**Cross-binding assays of HiR6.8 and HiR7.8. (A)** HiR6.8 binding to negative target cell lines used throughout screening and **(B)** cell lines not used as negative targets in screening. **(C)** HiR7.8 binding to negative target cell lines used throughout screening and **(D)** cell lines not used as negative targets in screening. Average values are graphed and normalized to target cells with errors bars representing standard deviations for three assays. * = p < 0.05, ** = p < 0.01, *** = p < 0.001.

Purified scFvs were each assayed for their ability to bind to cell lines other than LNCaP. These included the negative targets in the screening strategy, HGPIN, BPH-1, and BHPrE1 (Figure 5A & B). They also included androgen-independent prostate cancer cell lines DU-145 and PC-3 as well as normal prostate epithelium cell lines RWPE-1 and NHPrE1 (Figure 5C & D).From these experiments, it is clear that HiR6.8 binds to all three negative cell lines as well as the other four lines assayed (Figure 5A & B). Binding to many of these lines was as good or better than binding to LNCaP cells. This is a likely explanation as to why HiR6.8 was eliminated from both populations, in spite of its low dissociation constant. HiR7.8, however, showed excellent selectivity for LNCaP cells (Figure 5C & D). Binding to HGPIN, BPH-1, and BHPrE1 cells all were much less than to the target cancer cells. Subtraction of background secondary antibody MFI from scFv binding resulted in negative numbers due to binding of the secondary antibody to the scFv and not the cell lines. Additionally, binding to the four cell lines not used in screening and enrichment was significantly less relative to binding to target cells. In sum, this suggests that HiR7.8 is highly specific to androgen-dependent prostate cancer cells and therefore will be useful in both the therapeutic targeting and disease detection of prostate cancer.

## Discussion

The library screening and enrichment strategy used in this work focused on multiple, stringent negative enrichment targets to identify a scFv specific for androgen-dependent prostate cancer cells. Three rounds of panning were performed to remove scFvs with very low target affinity or that bound to molecules highly expressed on other cell surfaces [27]. There was a large amount of diversity present in the library however, so a more stringent separation method, FACS, was employed [28]. Initial use of FACS would have been unsuccessful as prior work has shown the efficiency of FACS is poor at low concentrations of the binding population [29,30]. Thus, removal of non-binding molecules increased the concentration of binding scFv in the population, allowing successful FACS isolation due to its single-cell and quantitative nature, as has previously been described [30,31]. Additionally, the initial library size was much too great to efficiently analyze single cells in a reasonable time span. Thus, panning followed by FACS was utilized, and the strong enrichment of the identified random sample of the library validates this strategy.

From the obvious enrichment through seven rounds of screening (Figure 2), HiR7.8 was chosen as the most likely prostate cancer cell-specific antibody fragment and underwent further characterization. However, as a representative of scFvs present in earlier rounds of screening and to determine why it was outcompeted, HiR6.8 was also chosen for further characterization. It is also important to note that HiR6.8 is a full-length scFv, comprising both a heavy and light chain, with a predicted molecular weight of approximately 30.6 kDa for the scFv itself. HiR7.8 and HiR7.6, however, only contain a heavy chain, as they are truncated just before the chain linker. They have a predicted molecular weight of approximately 16.0 and 15.6 kDa, respectively, and have 81% sequence identity and 86% sequence similarity. This may be relevant *in vivo*, as it has been shown that smaller scFvs have greater tumor penetration ability than larger antibodies [32]. It is possible that truncated scFvs will suffer from decreased stability. Future work will assay stability in envisioned applications and, if necessary, perform mutagenesis to select stable variants [33] or insert it into the scaffold of a stable full antibody [34].

The dissociation constants for each of these scFvs are in the low-nanomolar range, which is similar to previous reports of scFvs isolated from similar screening strategies [35-37]. More importantly, it is in a similar range to other antibody fragments that have strong pharmacokinetic properties [38,39]. It is possible that further mutagenesis and screening may select for scFvs with higher affinity for the cell surface target [37,40].

It may be possible that fluorescent staining images represent cellular uptake of the scFv-secondary antibody complex due to the cytoplasmic pattern. This has previously shown to be possible and even likely with scFvs alone or in complex with other molecules [41-43]. If these scFvs are taken up by androgen-dependent prostate cancer cells, this may have an important role in the clinical application of these scFvs. The potential ability to use these scFvs as therapeutic targeting agents in the form of antibody-drug conjugates (ADCs) is enhanced by scFv internalization [44]. Potential uptake of the scFv by androgen-dependent prostate cancer cells will be explored in future studies.

Biologically, the selectivity of HiR7.8 for androgen-dependent prostate cancer cells is important. HiR6.8 clearly binds to other cell lines tested, and this is likely the cause of its depletion from the library as the stringency of FACS enrichment increased. This depletion seems to validate the powerful selectivity of the FACS screening strategy. There is an absence of binding to all benign cell lines studied, whether they represent normal prostatic cells, BPH, or PIN. This suggests that the cell surface molecule HiR7.8 is binding to is not displayed on the surface of benign prostatic cells. This is supported by previous studies that have identified many genes differentially expressed between the benign conditions represented here and prostate cancer cells [45-47]. Additionally, there is no binding to the androgen-independent prostate cancer cell lines DU-145 or PC-3. This suggests that the cell surface antigen is displayed in the earlier, androgen-dependent stage of prostate cancer. This is possible considering previous studies have found a wide array of genes that are differentially expressed from androgen dependent to independent disease progression, many of which are downregulated [48-50]. In fact, downregulation of expression levels from LNCaP to both DU-145 and PC-3 cells has been found before [51-54] Future work will determine binding to other androgen-dependent prostate cancer cells utilizing antigen capture techniques coupled with mass spectrometry. Determining binding will be aided by identifying the exact cell surface molecule to which HiR7.8 is binding, as has been done before with similar cell surface antigen-binding molecules [55,56]. This type of work has even identified novel cell surface molecules previously unknown to be involved in disease progression [57]. It is therefore possible that this work not only created a novel targeting agent, but also identified a novel target.

The high affinity and specificity of HiR7.8 for androgen-dependent prostate cancer cells suggest it has potential in future therapeutic and diagnostic applications in prostate cancer. Though not determined here, future work will determine its binding to tissue representing androgen-dependent prostate cancer and other prostatic diseases which will determine the translational potential of the molecule. It is likely, however, that HiR7.8 will bind to androgen-dependent prostate cancer tissue from patients based on binding specificity and affinity similar to previously-identified molecules [58]. The selective scFv will likely be useful for drug delivery as has been shown before [59-62]. It has previously been noted that scFvs have much less immunogenicity than full antibodies in part due to their size and lack of an Fc component [63-65]. Additionally, it will likely have use in specific diagnosis of prostate cancer whether *in* or *ex vivo*[66-69]. Finally, identification of the cell surface markers to which these scFvs bind may possibly identify novel proteins, new functions, or expression patterns that aid in treatment and diagnosis of prostate cancer and will be done in future work. This cell surface molecule and its expression pattern in androgen-dependent prostate cancers will determine the translation of the identified scFv beyond the cells used here. While it is likely to be useful in a defined set of prostate cancers, the antibody fragment screening strategy described here also serves as a proof-of-principle for selection of other disease-specific antibody fragments.

## Conclusions

This work has selected a scFv antibody fragment, HiR7.8, with high affinity for androgen-dependent prostate cancer cells. Furthermore, the selected molecule has a high selectivity for the target cells and not for benign prostatic cells or for androgen-independent prostate cancer cells. These characteristics will allow the selected scFv to be useful for both therapeutic and diagnostic applications in the clinical treatment of prostate cancer with further pre-clinical development. With targeted therapeutics and more specific diagnostics, it is possible that increased efficacy and reduced side effects of treatments as well as earlier disease detection will be realized by the scFv obtained here.

## Methods

A library screening and enrichment process was used in order to obtain an androgen-dependent prostate cancer cell-specific antibody fragment (Figure 1). A library of non-immune human single-chain Fragment variable (scFv) antibody fragments, displayed on the surface of *Saccharomyces cerevisiae,* was a generous gift from Dr. Dane Wittrup (Massachusetts Institute of Technology; Cambridge, MA) [23]. Seven rounds of screening were completed, enriching for those scFvs which bound to androgen-dependent prostate cancer cells and subtracting those that bound to benign prostate cell lines as well as the protein PSMA.

### Cell culture and materials

In order to obtain a prostate cancer cell-specific scFv, prostatic cell lines were used. For general maintenance, each line was passaged every 5–7 days in a T75 cell culture dish with media changed every 2–3 days. The cells were grown in a 37°C incubator with 5% carbon dioxide and humidity. The LNCaP cell line was used as a model of androgen-dependent prostate cancer and was the target of positive enrichment. It was obtained from the American Type Culture Collection (ATCC) (Manassas, VA) and cultured in RPMI 1640 with L-Glutamine and 25 mM HEPES (Cellgro; Manassas, VA) and 10% Fetal Bovine Serum (FBS) (Fisher Scientific; Pittsburgh, PA) and 1X antibiotic/antimycotic mixture (ab/am) (Cellgro) [70]. The High Grade Prostatic Intraepithelial Neoplasia (HGPIN) cell line was a generous gift from Dr. Mark Stearns (Drexel University; Philadelphia, PA) and was cultured in Defined KSFM (Gibco; Grand Island, NY) with 5% FBS and 1X ab/am [71]. The Benign Prostate Hyperplasia (BPH-1) cell line was a generous gift from Dr. Simon Hayward (Vanderbilt University; Nashville, TN) and was cultured in RPMI-1640 with L-Glutamine and 25 mM HEPES and 10% FBS and 1X ab/am [72]. The intermediate prostate stem cell line BHPrE1 was also a generous gift from Dr. Simon Hayward and cultured in DMEM/F12 (Cellgro) supplemented with 5% FBS, 1X ab/am, 1% insulin/transferrin/selenium (Gibco), 0.4% bovine pituitary extract (Sigma; St. Louis, MO), 5 ng/mL epidermal growth factor (Gemini Bio-Products; West Sacramento, CA), and 1X ab/am [73]. The androgen-independent DU-145 prostate cancer cell line was obtained from ATCC and cultured in EMEM (Cellgro) with 10% FBS and 1X ab/am [74]. The androgen-independent prostate cancer cell line PC-3 was also obtained from ATCC and cultured in F12K media (Cellgro) with 10% FBS and 1X ab/am [75]. The normal prostatic epithelium cell line RWPE-1 was obtained from ATCC and cultured in Defined KSFM (Gibco) plus 1X ab/am [76]. The early prostate stem cell line NHPrE1 was a generous gift from Dr. Simon Hayward (Vanderbilt University) and cultured in the same media as BHPrE1 [73].

### scFv library and growth

A human non-immune scFv library with 10^9^ diversity displayed on the surface of *Sacchoromyces cerevisiae* was utilized [23,28]. The yeast library was chosen due to its amenability to FACS screening and the ability of yeast to display post-translationally modified proteins due to their eukaryotic nature. The library was amplified and expression induced as previously described [23,28]. Before each screening incubation, expression was verified by tagging with a monoclonal anti-HA tag antibody conjugated to either DyLight 488 (Columbia Biosciences; Columbia, MD) or AlexaFluor 488 (Invitrogen; Grand Island, NY). The samples were run on either a Cell Lab Quanta SC (Beckman Coulter; Brea, CA) or a FACSCalibur (BD Biosciences; San Jose, CA) flow cytometer equipped with a 488 nm argon laser and 525 nm emission filter.

### Library screening

Seven rounds of screening were performed in order to obtain a scFv specific for androgen-dependent prostate cancer cells (Table 1). The first three rounds of screening were performed by panning and the last four by fluorescence-activated cell sorting (FACS).

For Round 1(+) screening, androgen-dependent LNCaP prostate cancer cells were grown to 80-90% confluency and the media was removed. The cells were gently washed with calcium- and magnesium-free phosphate-buffered saline (PBS). The cells were then incubated with 10^10^ yeast from the naïve library in 15 mL yeast screening buffer (YSB) containing PBS, 0.5% bovine serum albumin (BSA) and 1% FBS. The library was placed into the flask containing prostate cells and placed on a 37°C shaker at 25 RPM for three hours. After incubation, yeast not bound to the cells were removed, and the LNCaP cells were gently washed three times with 15 mL YSB and confluence of remaining attached cells was visually confirmed. 100 mL yeast amplification media was added to the flask to allow for amplification of yeast bound to the prostatic cells. This was grown overnight, and this enriched library was prepared for a round of negative enrichment. For Round 1(−) screening, scFv-expressing yeast were suspended in YSB and incubated with rinsed HGPIN cells at 80-90% confluency for 30 minutes at 37°C with shaking at 25 RPM. The supernatant containing yeast-displayed scFvs not bound to HGPIN cells was removed and added to rinsed BPH cells under the same conditions, removed again and added to rinsed normal prostatic epithelium BHPrE1 cells under the same conditions. The serially-incubated supernatant was centrifuged to obtain yeast, which were amplified. Two more rounds of panning were performed in this manner, with decreasing incubation times for positive rounds and increasing incubation times for negative rounds.

The ensuing four rounds of screening were performed using FACS-based separation (Additional file 1: Figure S1). For Round 4(+), LNCaP cells grown to 80-90% confluency were fluorescently dyed with CFSE (Invitrogen) according to manufacturer’s instructions. They were then dissociated from the flask with Cellstripper reagent (Cellgro) to prevent cell surface protein digestion associated with trypsinization. Cells were then suspended in YSB and counted with a Scepter equipped with 60 μm sensors (Millipore; Billerica, MA). In Round 4, all yeast were fluorescently dyed with Syto61 (Invitrogen) according to manufacturer’s instructions. A total of 10^7^ yeast were suspended in YSB and mixed with 10^6^ LNCaP in 2.5 mL. They were mixed by inversion for 30 minutes at 37°C and placed on ice before FACS sorting. The sample was then sorted with a FACSAria (BD Biosciences), with excitation at 488 nm from a sapphire solid state laser and 633 nm from a HeNe laser and 525 nm and 650 nm emission filters. Events identified as bound yeast and prostate cells were collected. In Round 4(+), there were two separate populations of bound cells which were collected and amplified separately. These were named the ‘Hi’ and ‘Lo’ populations and kept separate through the following rounds of screening and subjected to the identical conditions.

In Round 4(−)a, for each of the Hi and Lo populations, yeast were prepared for screening in the same manner and incubated with 10^6^ HGPIN cells dyed the same with inversion for 30 minutes at 37°C in YSB. The sample was subjected to FACS and events that indicated yeast that were not bound to HGPIN cells were collected and amplified. Those were then incubated with 10^6^ dyed BPH cells for Round 4(−)b and subjected to FACS with unbound yeast collected and amplified. The enriched library was then prepared for Round 4(−)c and subjected to FACS after incubation with 10^6^ dyed BHPrE1 cells. Unbound yeast were collected and amplified, then prepared for screening and incubated with 1 nmol recombinant full-length PSMA protein, which is expressed in non-prostatic normal and tumor tissue, as well as normal prostate cells [77] (Abnova #H00002346-P01; Walnut, CA). This protein is 107 kDa and 719 amino acids with a GST tag and was pre-incubated with AlexaFluor 488-conjugated anti-GST tag antibody (Invitrogen). The yeast which showed single fluorescence signals corresponding to Syto61 were kept and amplified. Positive enrichment rounds continued through Round 7, with yeast fluorescent tagging being done with anti-HA tag antibody conjugated to AlexaFluor 647 (Invitrogen) with decreasing target cell concentrations. This tagging method was used for a negative enrichment performed in Round 6.

### Sequencing of scFv library

A representative sample of scFv genes from the naïve library (Round 0) was sequenced to determine diversity of the library. Additionally, this was completed for the enriched post-Round 2(−) and post-Round 3(−) libraries. It was also performed for each of the Hi and Lo populations following Round 4(−), Round 6(−) and Round 7(+). To do this, yeast were plated onto amplification media agar, and individual colonies were chosen for polymerase chain reaction (PCR) amplification.

Yeast colonies were picked and placed into double distilled water and boiled, which served as template for the PCR reaction. The reaction ingredients were as follows: 400 nM forward (5′-GTACGAGCTAAAAGTACAGTG-3′) and reverse (5′-TAGATACCCATACGACGTTC-3′) pPNL6 primers (Eurofins MWG Operon), 250 μM deoxynucleotide triphosphates, 5% dimethyl sulfoxide, 1X Phusion Reaction Buffer (New England Biolabs, Ipswich, MA), 2 units Phusion High-Fidelity DNA polymerase (New England Biolabs), and double distilled water to 100 μL. Reactions conditions were: initial denaturation at 98°C for 30 seconds; 35 cycles of 98°C for 10 seconds, 50°C for 30 seconds, and 72°C for 30 seconds; and final extension at 72°C for 10 minutes. Results were analyzed using agarose gel electrophoresis and those PCRs that contained bands corresponding to the scFv gene were purified using a PCR purification kit (IBI Scientific; Peosta, IA) and sent for DNA sequencing (Eurofins MWG Operon; Huntsville, AL) using both the forward and reverse pPNL6 primers. In total, 30–35 sequences were obtained for each enriched library noted above, including for individual Hi and Lo populations separately. Analysis was then performed by translating the DNA sequence to protein using the ExPASy translate tool (Swiss Institute of Bioinformatics; http://web.expasy.org/translate/). Hemagluttanin, c-Myc, and linker protein tag sequences, landmarks of the scFv expression scaffold, were identified to ensure sequence quality [23]. Sequences were compared for similarities and duplicates within an enriched library were identified to determine diversity of a random sample of the library.

### Secretion and purification of selected scFvs

From the Rounds 6(−) and 7(+) sequences, one scFv, HiR7.8, was chosen for further study due to its abundance in the enriched libraries. HiR6.8 was also chosen to be representative of scFvs present earlier in the screening process and to determine why it was outcompeted from the population by HiR7.8. These sequences were subcloned into the pPNL9 secretion vector in YVH10 yeast using gap repair essentially as previously described [28]. For truncated scFv sequences, a modified reverse PCR primer (5′- GGGTTAGGGATAGGCTTACCGAACTCTGAAGAGACGGTGACC-3′) was used. After growth of the yeast containing the scFv sequence within the secretion vector, the supernatant was recovered and scFv purified with Ni-NTA resin following the manufacturer’s protocol (Thermo Scientific; West Palm Beach, FL). Purified scFv was analyzed by polyacrylamide gel electrophoresis as previously reported, and the concentration of the scFv was determined using spectroscopy with a NanoDrop (Thermo Scientific). The scFv was then diluted to a working concentration of 10 μM in PBS.

### Affinity binding assays with scFvs

In order to determine the binding affinity of the secreted scFvs with the target androgen-independent prostate cancer cells, saturation binding assays were performed essentially as previously described [36,40]. LNCaP cells were grown to 80-90% confluency and removed from the flask with CellStripper reagent. They were counted and 2 × 10^5^ cells were placed into a 500 μL total volume of YSB. For saturation binding assays, the appropriate amount of scFv was incubated on ice for 30 minutes with 2 μL of the monoclonal anti-HA antibody conjugated to DyLight 488 (Columbia Biosciences). Concentrations of 0, 1, 10, 100, 1000, 10000, 50000, 100000, 150000, 200000 pM of the scFv were used. The pre-incubated scFv and secondary antibody were mixed with LNCaP cells and placed on a rotisserie at 37°C for 30 minutes. After this period, the cells were washed and run on a FACSCalibur (BD Biosciences) as previously described. The mean fluorescence intensity (MFI) for each sample was recorded. The MFI for the 0 pM incubation, which had only the secondary fluorescent antibody, was subtracted from the MFI of each incubation. These data were graphed using Origin 8 software (OriginLab Corporation; Northampton, MA). The data were fit with a nonlinear regression model of single-event binding given by the equation, Y = ((B_max_*X)/(K_d_ + X)) + NS*X, where B_max_ is maximum binding, K_d_ is the dissociation constant, and NS is nonspecific binding [78]. Each assay was performed in triplicate, with the K_d_ values averaged and the standard errors of the Origin-obtained K_d_ values were combined.

### Fluorescent imaging of scFv binding

In order to visualize binding of each scFv, fluorescent images were taken. Target androgen-independent prostate cancer cells were grown in a 6-well culture dish on number 1.5 microscopic cover glasses to ~90% confluency. For each secreted scFv, a 100 nM concentration was pre-incubated with anti-HA antibody conjugated to DyLight 488 as described for binding assays. Cells incubated with anti-PSMA AlexaFluor488 antibody served as a positive control (BioLegend; San Diego, CA) and cells incubated with only anti-HA DyLight 488 antibody served as negative control. Cells were incubated at 37°C for 30 minutes with shaking at 25 RPM. The supernatant was removed and the cells were washed, formalin-fixed, and mounted onto microscope slides. Cells were imaged on a Zeiss AxioImager Z2 Fluorescent Microscope (Zeiss; Thornwood, NY) using an Argon laser at 488 nm for excitation with a band pass emission filter at 505–530 nm.

### Specificity binding assays of scFvs

In order to determine the specificity of scFv binding, cross-binding assays were performed with the cells used as negative targets. LNCaP, HGPIN, BPH-1, and BHPrE1 cells were counted and 2 × 10^5^ cells were placed into 500 μL YSB. Concentrations of 0 or 100 nM scFv were incubated with the secondary anti-HA AlexaFluor 488 antibody for 30 minutes on ice. Cell were prepared as described above and then run on a FACSCalibur and the MFI recorded. The MFI of the 0 pM incubation, which had only the secondary fluorescent antibody, served as background fluorescent labeling and was subtracted from the 100 nM incubation. Data were normalized to target cell binding, which was set to 100%, and graphed to show comparative binding between the cell lines.

In order to determine binding to other prostatic cell lines not used throughout screening, cross-binding assays were performed. The target androngen-dependent prostate cancer cells, the androgen-independent prostate cancer cell lines DU-145 and PC-3, and the normal prostatic cell lines RWPE-1 and NHPrE1 were used for this experiment. The cells were prepared as noted above and run on a FACSCalibur. Each set of cross-binding assays were performed in triplicate and significance in the differences of means were obtained using a student’s *T*-test.

## Competing interests

The authors declare no commercial or financial conflict of interests.

## Authors’ contributions

RMW conceived of the manuscript and designed all experiments, performed the experiments, analyzed the data, and drafted the manuscript. CJH and SN performed experiments and analyzed the data. LJS conceived of the manuscript and designed all experiments, analyzed the data, and drafted the manuscript. All authors read and approved the manuscript.

## Supplementary Material

Additional file 1: Figure S1 Representative FACS plots from the selection. For all, the X-axis is CFSE fluorescence, which was used to stain the target cell line and the Y-axis is Syto 61 or Alexa647 (anti-HA antibody) which was used to stain yeast expressing the library. Q1 shows stained yeast alone, Q2 shows events representing yeast bound to the target cell line, Q3 shows stained cells alone, and Q4 shows unstained cells and debris. **A)** FACS plot from Round 4(+) selection with the target LNCaP cell line. The origination of the “Hi” and “Lo” populations are shown and so named due to amount of yeast staining present in the events. **B)** FACS plots from Round 5(+) selection with the target LNCaP cell line. Left shows sorting of the yeast binding to the LNCaP cell line in the Lo population and right shows sorting of the yeast binding to the LNCaP cell line in the Hi population. **C)** FACS plots from Round 6(-)c selection with the non-target BHPrE1 cell line. Left shows sorting of yeast that did not bind to BHPrE1 cells in the Lo population and right shows sorting of yeast that did not bind to the BHPrE1 cells in the Hi population. Click here for file
