# Supplementary material for: Identification of an antibody fragment specific for androgen-dependent prostate cancer cells
Source: BMC Biotechnol. 2014 Sep 3;14:81. doi: 10.1186/1472-6750-14-81 (PMC4158339; doi:10.1186/1472-6750-14-81)
Supplement: Additional file 1: Figure S1 — Representative FACS plots from the selection. For all, the X-axis is CFSE fluorescence, which was used to stain the target cell line and the Y-axis is Syto 61 or Alexa647 (anti-HA antibody) which was used to stain yeast expressing the library. Q1 shows stained yeast alone, Q2 shows events representing yeast bound to the target cell line, Q3 shows stained cells alone, and Q4 shows unstained cells and debris. A) FACS plot from Round 4(+) selection with the target LNCaP cell line. The origination of the “Hi” and “Lo” populations are shown and so named due to amount of yeast staining present in the events. B) FACS plots from Round 5(+) selection with the target LNCaP cell line. Left shows sorting of the yeast binding to the LNCaP cell line in the Lo population and right shows sorting of the yeast binding to the LNCaP cell line in the Hi population. C) FACS plots from Round 6(-)c selection with the non-target BHPrE1 cell line. Left shows sorting of yeast that did not bind to BHPrE1 cells in the Lo population and right shows sorting of yeast that did not bind to the BHPrE1 cells in the Hi population. [file 1472-6750-14-81-S1.docx]

**Identification of an Antibody Fragment Specific for Androgen-Dependent Prostate Cancer Cells**

**Ryan M. Williams^1,2^^, Cyrus J. Hajiran^3^, Sara Nayeem^1^, and Letha J. Sooter^1^***

**^1^ West Virginia University, Department of Basic Pharmaceutical Sciences, 1 Medical Center Drive, PO Box 9530, Morgantown, WV 26506**

**^2^ Current address: Memorial Sloan Kettering Cancer Center, Molecular Pharmacology & Chemistry Program, 1275 York Ave., New York, NY 10065**

**^3^ West Virginia University, Department of Biology, 53 Campus Drive, PO Box 6057, Morgantown, WV 26506**

**^^^ Pre-publication Correspondence Email: rwilliams@mix.wvu.edu; Phone: (646) 888-3416**

***Post-publication Corresponding Author Email: lsooter@hsc.wvu.edu; Phone: (304) 293-9218**

**Supplementary Information**

**Supplementary Figure**

**
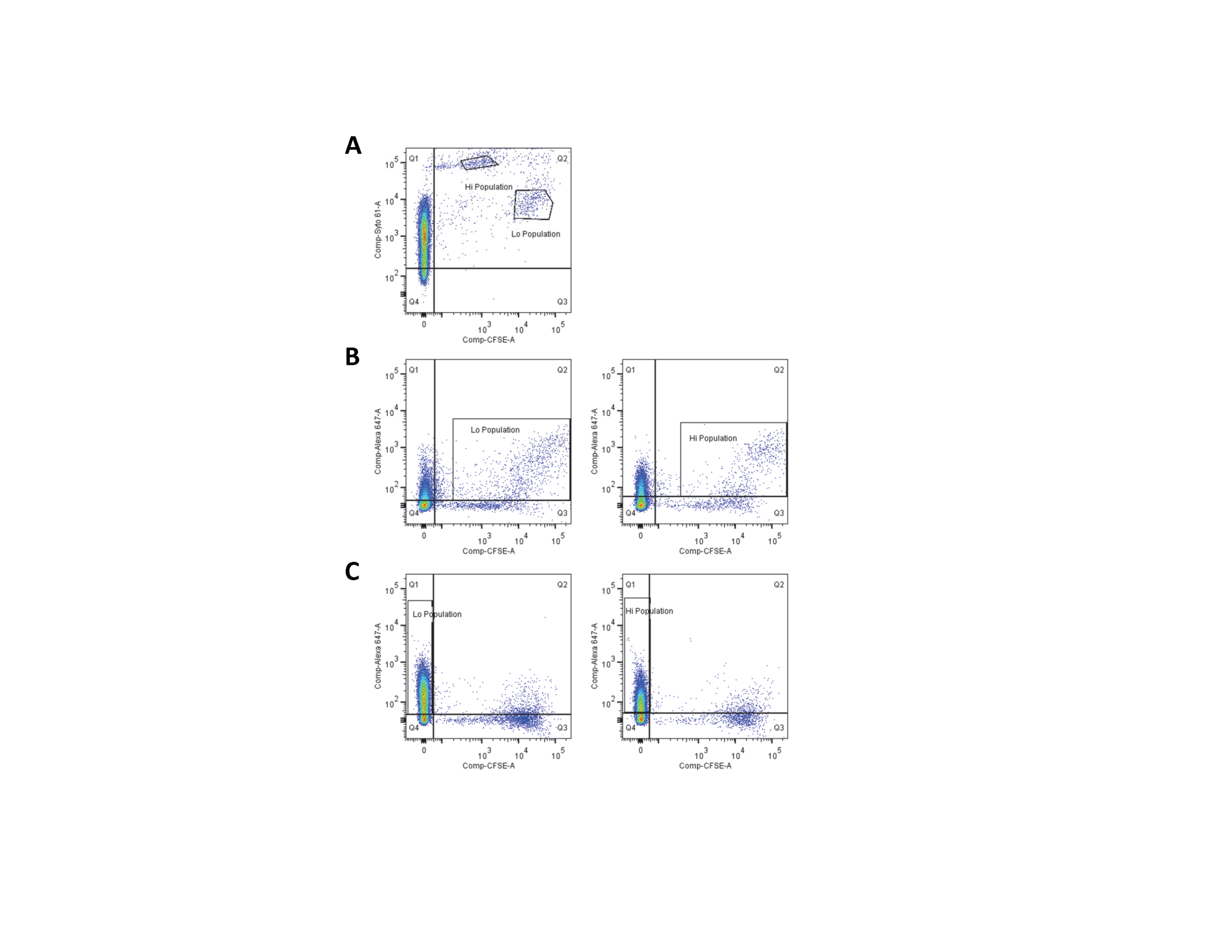
**

**Figure S1. Representative FACS plots from the selection.** For all, the X-axis is CFSE fluorescence, which was used to stain the target cell line and the Y-axis is Syto 61 or Alexa647 (anti-HA antibody) which was used to stain yeast expressing the library. Q1 shows stained yeast alone, Q2 shows events representing yeast bound to the target cell line, Q3 shows stained cells alone, and Q4 shows unstained cells and debris. **A)** FACS plot from Round 4(+) selection with the target LNCaP cell line. The origination of the “Hi” and “Lo” populations are shown and so named due to amount of yeast staining present in the event. **B)** FACS plots from Round 5(+) selection with the target LNCaP cell line. Left shows sorting of the yeast binding to the LNCaP cell line in the Lo population and right shows sorting of the yeast binding to the LNCaP cell line in the Hi population. **C)** FACS plots from Round 6(-)c selection with the non-target BHPrE1 cell line. Left shows sorting of yeast that did not bind to BHPrE1 cells in the Lo population and right shows sorting of yeast that did not bind to the BHPrE1 cells in the Hi population.
